# Delay Analysis of Networked Control Systems Based on 100 M Switched Ethernet

**DOI:** 10.1155/2014/751491

**Published:** 2014-06-05

**Authors:** Ming Li

**Affiliations:** College of Mathematics & Computer Science, Wuhan Textile University, Wuhan 430200, China

## Abstract

For the delay may degrade the performance of networked control systems, networked control systems based on 100 M switched Ethernet are proposed in this paper. According to the working principle of Ethernet switch, the formulas of the upper bound delay of the single-level switched Ethernet and the multiple-level switched Ethernet are deduced by the timing diagram method, and the values of the upper bound delay are also given. The key factors that influence the upper bound delay of switched Ethernet are analyzed; then, the characteristics of the upper bound delay are presented, which show that the delay induced by the single-level 100 M switched Ethernet has little effect on the performance of control systems, while the delay induced by the multiple-level 100 M switched Ethernet may meet the time requirements of all classes of control systems if the numbers of levels and the numbers of nodes connecting to switches are set properly. Finally, the performance of networked control systems is simulated by TrueTime, and the results further show the feasibility and superiority of 100 M switched Ethernet based networked control systems without modification of the network protocols.

## 1. Introduction

Networked control systems (NCSs) are feedback control systems wherein the control loops are closed through real-time control networks. For NCSs use the public or private communication networks to replace the point-to-point data transmission of traditional control systems, they have the advantages of reduced system wiring, ease of system diagnosis and maintenance, and increased system agility [1, 2]. But because of the limited bandwidth and the QoS of the communication networks, there exist some problems, such as network-induced delay and packet dropouts, in the NCSs. These problems can degrade the performance of control systems and can even destabilize the control systems. Therefore, the control networks are one of the most important factors that affect the performance of the NCSs [2–6]. There are many people researching these problems of the NCSs. Kim et al.  [7] proposed a new scheduling method to obtain a maximum allowable delay bound for the scheduling of networked discrete control systems. Lian et al. [8] studied the key components of time delay to provide guidelines for obtaining the optimal working range of sampling times. Walsh et al.  [9] introduced a novel control network protocol, try-once-discard (TOD), for multiple-input-multiple-output NCSs. But these works cannot consider the usage of switched Ethernet in an NCS.

Switched Ethernet is one of the fastest growing LAN technologies nowadays, and it overcomes the limitation of shared Ethernet; therefore, it can significantly improve the real-time data transmission of control networks. Currently, the technology of 100 M switched Ethernet is very mature and is already used very widely [10]. Since switched Ethernet has many advantages over other fieldbuses, it has obtained more and more attention of researchers in the field of control [11–15]. But current researches are mainly focused on 10 M shared Ethernet, and the network protocols must be modified to some extent in order to obtain real-time data transmission; therefore, the conclusions obtained from 10 M shared Ethernet cannot be directly applied to 100 M switched Ethernet.

Aiming at the performance degradation of control systems caused by the network-induced delay, the 100 M commercial switched Ethernet is presented to establish an NCS without any modification of the network protocols in this paper. This paper is organized as follows. First, the upper bound delay of the 100 M commercial switched Ethernet with single level and multiple levels is analyzed by the timing diagram in order to show how the delay induced by switched Ethernet affects the performance of an NCS. Then, through a case, the tool TrueTime is used to simulate the performance of an NCS based on 100 M commercial switched Ethernet to show the feasibility and superiority of this class of NCSs. Finally, we present our conclusions.

## 2. Delay Description of an NCS

**Figure 1:**
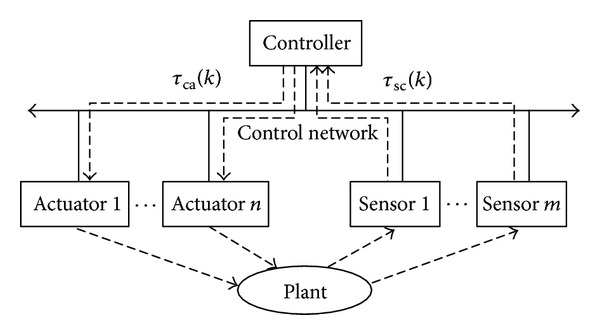
The structure of networked control systems.

In NCSs, many nodes, such as the sensors, actuators, and controllers, must use control networks to exchange the data to complete the control tasks, as shown in Figure 1  [1–4].

This delay caused by the network communication is called network-induced delay. There exist the delay from the sensors to the controllers *τ*_sc_(*k*) and the delay from the controllers to the actuators *τ*_ca_(*k*) in Figure 1, where *k* is the sampling serial number. At the sampling time *kT*, the whole delay induced by the control network can be denoted as *τ*_*k*_ = *τ*_sc_(*k*) + *τ*_ca_(*k*). The delay is one of the key problems when analyzing and designing an NCS  [16].

The delay of an NCS is actually induced in the communication process when the data are transmitted from one node to other nodes; in such process, the data are encapsulated in the source nodes and decapsulated in the destination nodes [6]. The delay induced in this process is made up of four parts: the processing delay for transmission at the source *T*_send_, the waiting delay *T*_wait_, the transmission delay *T*_ts_, and the processing delay for reception at the destination *T*_rev_, where *T*_send_ and *T*_wait_ are produced at the source node, *T*_rev_ is produced at the destination node, and *T*_ts_ is the sum of the transmission time of the data frames and the propagation time of the channel. Therefore, the whole delay *τ* can be denoted as

(1) $$\begin{array}{c} \tau =T_{\text{send}}+T_{\text{wait}}+T_{\text{ts}}+T_{\text{rev}}. \end{array}$$

The delay induced by control networks may be constant, time-variant, or random according to the communication protocols used by control networks; also, the delay may be limited or unlimited [17]. Shared Ethernet, for example, uses the protocol CSMA/CD, so the delay is random and unlimited, while the delay induced by CAN fieldbus is random and limited because of the protocol CSMA/BA.

The methods used to analyze and design an NCS may be different because of the different characteristics of the delay. The random delay,  for example,  can be changed into the fixed one when a buffer is used at the receiving node,  or the random delay can be modeled as Markov chain in order to simplify the analysis of an NCS. However, as for the delay based on the Internet, the dynamic model of the end-end delay can be established through the system identification. Moreover, an NCS can be analyzed and designed according to whether the delay is smaller or bigger than one sampling period.

The standard IEC 61784-2 made by the International Electric Committee classifies the industrial control systems into 3 classes according to the transmission time between two nodes [12]. We can use this standard to judge whether the network-induced delay meets the requirements of a control system.

1. *The First Class.* The delay is less than 100 ms. Most processes in process automation and building control fall into this class.
2. *The Second Class.* The delay is less than 10 ms. This is the requirement for most tooling machine control systems like programmable logic controllers (PLCs) or PC-based control.
3. *The Third Class.* The delay is less than 1 ms with a jitter of not more than 1 *μ*s. It is imposed by the requirement of motion control systems.

## 3. Upper Bound Delay of 100 M Switched Ethernet

An Ethernet switch can effectively identify the destination of data frames and relay the frames only to the destination ports without affecting other ports, so Ethernet switch can isolate the collision domain of the networks and suppress the broadcast storm. Ethernet switch can transmit the frames through three methods: cut-through, store-and-forward, and fragment-free [10].

**Figure 2:**
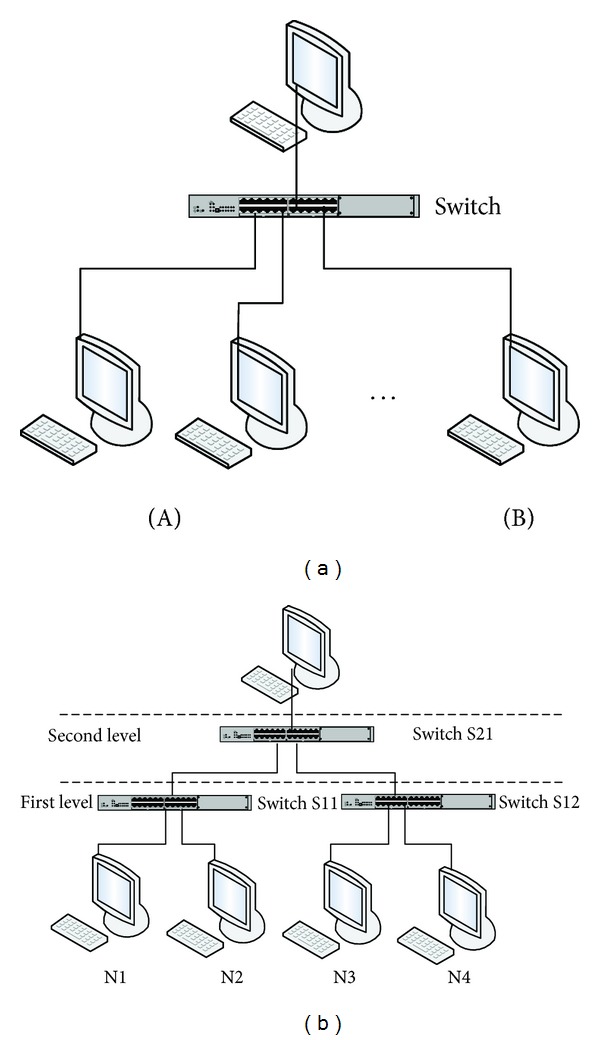
The hierarchical structure of switched Ethernet.

According to the connection mode between switches and nodes, the single-level switched Ethernet and the multiple-level switched Ethernet can be classified.  Figure 2(a) is a single-level switched Ethernet where only one switch exists in the whole networks; however, Figure 2(b) is a simple two-level switched Ethernet where there are three switches and two levels in the whole networks.

In order to analyze the upper bound delay of 100 M switched Ethernet, some necessary conditions should be made as follows.

1. The switching technology of a switch is store-and-forward.
2. The buffer of a switch is large enough and no packet overflows from the switch.
3. All the cables in the networks are of the same length.

### 3.1. Upper Bound Delay of Single-Level 100 M Switched Ethernet

A single-level 100 M switched Ethernet is suitable to establish an NCS that is located in a small range with a few controlled parameters and does not have complex control tasks. If the transmission medium is the twisted-pair cable, the single-level switched Ethernet will have the coverage area where the radium is 100 m and the switch is located in the center. Because there is only one switch in the network, every node must connect to a port of the switch.

**Figure 3:**
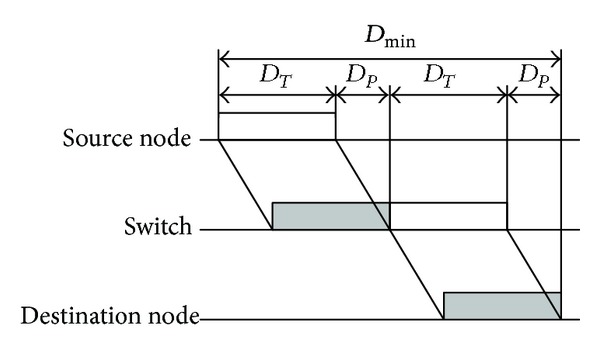
The timing diagram of the minimum delay of a single-level switched Ethernet.

When the source node A sends data to the destination node B, the timing diagram of the data from A to B is shown in Figure 3, provided that no data exist in the buffer of the switch.

According to Figure 3, the formula of the minimum delay is obtained as follows:

(2) $$\begin{array}{c} D_{\min}=2\left(D_{T}+D_{P}\right), \end{array}$$

where *D*_*T*_ is the transmission delay of the data frames and its value equals the length of the frame divided by the transmission rate and *D*_*P*_, proportional to the length of the communication media, is the propagation delay from the source to the destination.

If the 100 M switched Ethernet conforms to the standard 100BASE-TX, the value of the minimum delay can be calculated as follows (the length of the frame is set as 576 bits that is big enough to transmit control message):

(3) Dmin⁡=2(DT+DP)=2×(576100×106+1000.65×3×108)=12.55 μs.

So the minimum delay of the single-level 100 M switched Ethernet is very small compared with the requirements of all classes of control systems, and then it can be ignored completely.

**Figure 4:**
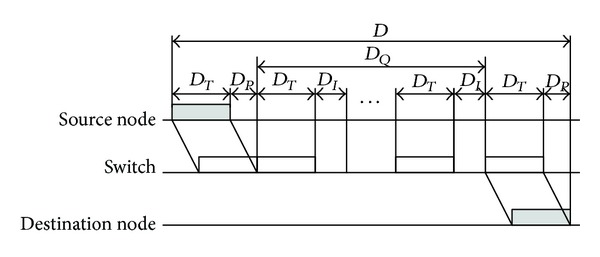
The timing diagram of a single-level switched Ethernet.

If there are some data in the buffer of a switch, the timing diagram of the data from A to B is shown in Figure 4. At this situation, the delay *D* will be the sum of *D*_min⁡_ and queuing delay *D*_*Q*_; that is,

(4) $$\begin{array}{c} D=D_{\min}+D_{Q}. \end{array}$$

Supposing that there are *N*_*Q*_ frames in the buffer of the switch, the queuing delay *D*_*Q*_ can be calculated as

(5) $$\begin{array}{c} D_{Q}=\overset{N_{Q}}{\underset{K=1}{\sum }}(D_{I}+\max\left(L_{k}+L_{h},576\right)\times t_{b}), \end{array}$$

where *D*_*I*_ (defined as 0.96 *μ*s) is the interframe delay; *L*_*k*_ is the length of valid data in the *k*_th_ frame; *L*_*h*_ is the overhead of a frame and is defined as 26 bytes here; and, also, *t*_*b*_ denotes the bit time that is defined as 0.01 *μ*s in the 100 M switched Ethernet.

Obviously, the upper bound delay (*D*_up_) of the single-level switched Ethernet will be obtained when the numbers of the data frames (*N*_*Q*_) in the buffer of the switch reach the maximum.

If there are *N*_*s*_ nodes connecting to a switch and *N*_*s*_ − 1 nodes simultaneously send data to one node, the maximum value of *N*_*Q*_ will be reached, that is, *N*_*s*_ − 1. So the formula of the upper bound delay of the single-level switched Ethernet should be shown as follows:

(6) Dup=Dmin⁡+(Ns−1)×(DI+max⁡(LK+Lh,576)×tb)=12.55+(Ns−1)×(0.96+max⁡(LK+Lh,576)×tb).

In order to illustrate this formula, *N*_*s*_ is assumed to be equal to 141. Therefore, the upper bound delay can be calculated as

(7) $$\begin{array}{c} D_{\text{up}}=12.55+140\times \left(0.96+5.76\right)=953.55\;\mu \text{s}. \end{array}$$

From the above value, we can see that the upper bound delay of the single-level 100 M switched Ethernet is far less than the requirements of motion control systems even though there are a large number of data frames in the buffer of a 100 M switch. In fact, it is very rare that there exist 140 frames in the buffer of a 100 M switch if the network is in the stable situation.

Now, the characteristics of the single-level 100 M switched Ethernet can be concluded as follows: the upper bound delay is only affected by the numbers of the data frames in the buffer of a switch and has nothing to do with the network loads; more than one NCS can be established by only one 100 M Ethernet switch; and each NCS has no effect on others if the source port and the destination port in each control loop are completely different. It is feasible to apply the single-level 100 M switched Ethernet to all classes of NCSs.

### 3.2. Upper Bound Delay of Multiple-Level 100 M Switched Ethernet

A multiple-level 100 M switched Ethernet is formed by more than one switch to cover relatively wider area. The advantage of the multiple-level switched Ethernet is that it can easily establish the hierarchical and distributed control systems, which is helpful to the NCSs that have a lot of controlled parameters and have very complex control tasks.

**Figure 5:**
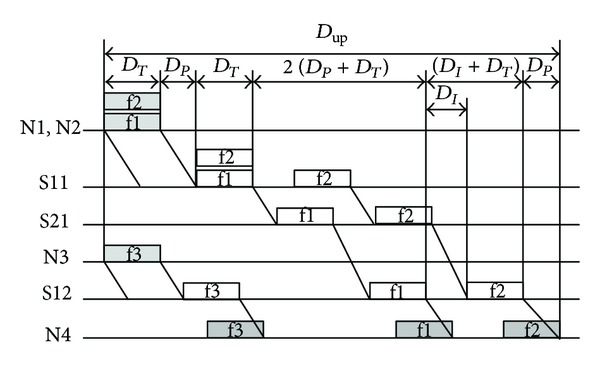
The timing diagram of a two-level switched Ethernet.

In order to obtain the upper bound delay of the multiple-level 100 M switched Ethernet, let us firstly analyze a simple two-level switched Ethernet as shown in Figure 2(b). The first level of the two-level switched Ethernet has two 100 M switches (S11 and S12) and each switch is connected by two nodes; the second level has only one 100 M switch (S21) that is connected by two first-level switches. When three nodes (N1, N2, and N3) send data to the node N4 simultaneously, the upper bound delay (*D*_up_) will be produced. At this situation, the timing diagram of the two-level switched Ethernet is shown as in Figure 5.

The communication process can be described as follows. Firstly, the data frames f1 and f2 will reach the switch S11 simultaneously, while the data frame f3 will reach the switch S12 according to its road. Then, the data frame f1 (or f2) will be transmitted to the switch S21 through the switch S11, while the data frame f2 (or f1) will wait to be transmitted in the buffer of the switch S11 until f1 (or f2) is finished, but the data frame f3 will be directly transmitted to the node N4 through the switch S12. Finally, the data frame f1 will reach the node N4 through the switch S12 and then the data frame f2 will also reach the node N4 after an interframe delay between two successive frames. At this situation, the maximum number of the switches from the source to the destination is three, and the maximum number of the frames in the buffer of the switch from the source to the destination is two.

So the upper bound delay of the NCS can be calculated as

(8) Dup=Dmin⁡+(3−1)(DP+DT)+(2−1)(DI+DT)=12.55+2×6.273+6.72=31.82 μs.

In general, let us assume that the number of the levels is *N*_*L*_, the maximum number of the nodes connecting to one switch is *N*_*s*_, the maximum number of the switches from source nodes to destination nodes is *N*_*E*_, and the maximum number of the frames in the buffer of a switch is *N*_*Q*_′; then, the following can be obtained:

(9) $$\begin{array}{c} N_{E}=2N_{L}-1,\;\;N_{Q}^{\prime }=N_{s}. \end{array}$$

Therefore, the formula of the upper bound delay of multiple-level switched Ethernet is given as follows:

(10) Dup=Dmin⁡+(NE−1)(DP+DT)+(NQ′−1)(DI+DT)=Dmin⁡+(2NL−2)(DP+DT)+(NS−1)(DI+DT).

From the formula (10), we can see that the key factors are *N*_*L*_ and *N*_*S*_ that affect the upper bound delay of multiple-level switched Ethernet.

For example, if there are 3500 parameters that need to be measured and controlled, an NCS based on the multiple-level 100 M switched Ethernet is designed that has about 600 sensor nodes and 500 actuator nodes. Let us consider the following two topologies.

(1) The *N*_*L*_ is set as two and the *N*_*S*_ is set as ten. Then, the upper bound delay can be calculated as
  (11) Dup=12.55+(3−1)(0.513+5.76)+(10−1)(0.96+5.76)=85.58 μs.
  We can see that the value is very small and satisfies the time requirements of all classes of control systems.
(2) The *N*_*L*_ is set as eight and the *N*_*S*_ is set as one hundred forty. Then, the upper bound delay can be calculated as
  (12) Dup=12.55+(15−1)(0.513+5.76) +(140−1)(0.96+5.76)=1034.452 μs.
  At this situation, the upper bound delay does not satisfy the requirement of motion control systems, but it still satisfies the requirements of the first class and the second class of control systems.

Now, the characteristics of the upper bound delay of the multiple-level 100 M switched Ethernet can be concluded as follows: the upper bound delays of the multiple-level 100 M switched Ethernet under different network topologies are different from each other. If the numbers of levels and the numbers of nodes connected to the switches in the networks are set properly, the delay induced by the multiple-level 100 M switched Ethernet can meet the time requirements of all classes of control systems.

## 4. Simulation Analysis

In order to further illustrate the effect of the delay induced by 100 M switched Ethernet on the performance of an NCS, consider the following controlled plant:

(13) [x˙1x˙2]=[3−210][x1x2]+[10]u,y=[01][x1x2].

**Figure 6:**
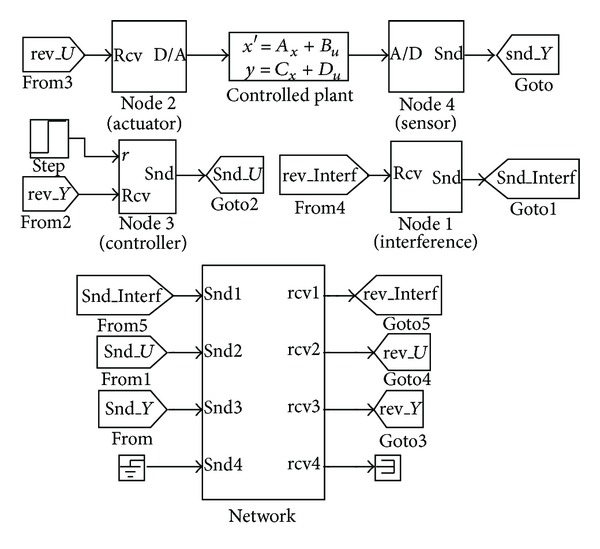
The simulation diagram of the NCS based on single-level 100 M switched Ethernet.

An NCS is established by 100 M commercial switched Ethernet in order to control the plant, where the PID control method is used, that is, proportional coefficient *K*_*P*_ = 500, integral coefficient *K*_*I*_ = 30, and differential coefficient *K*_*D*_ = 30, and the sampling period is set as 8 ms. The TrueTime tool [18–22] is adopted to simulate the performance of the NCS under some different network environments, where the sensor nodes are clock-driven, the controller nodes and actuator nodes are event-driven, and all the nodes on the networks use the strategy of prioFP and have the same priority.  Figure 6 is the simulation diagram of the NCS based on a single-level 100 M switched Ethernet.

**Figure 7:**
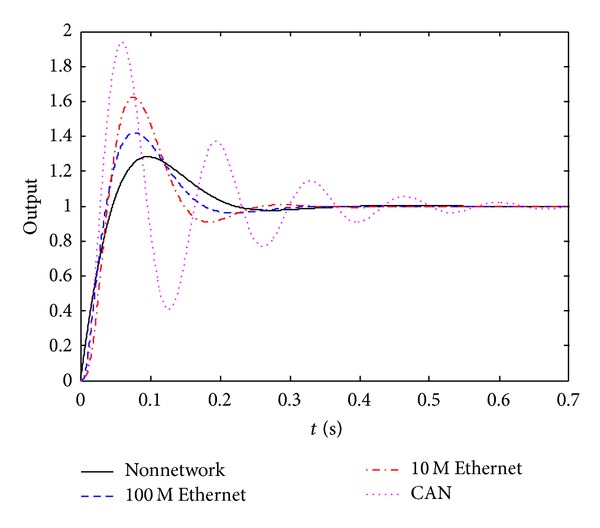
The step responses of the control systems under different network environments.

In order to compare the performance of the control systems under different network environments, 10 M switched Ethernet, CAN fieldbus, and nonnetwork environment are also used here. The step responses of the control systems under different network environments can be obtained as Figure 7 shows.

The performance indexes of different control systems are shown in Table 1. We can find that the performance of the NCS based on single-level 100 M switched Ethernet is very similar to that of the nonnetworked control system, and their performances are more better than those of the NCS based on 10 M switched Ethernet and CAN fieldbus. Moreover, the control strategy and the control method of the nonnetworked control system can be used directly by the NCS based on single-level 100 M switched Ethernet without any modification.

**Figure 8:**
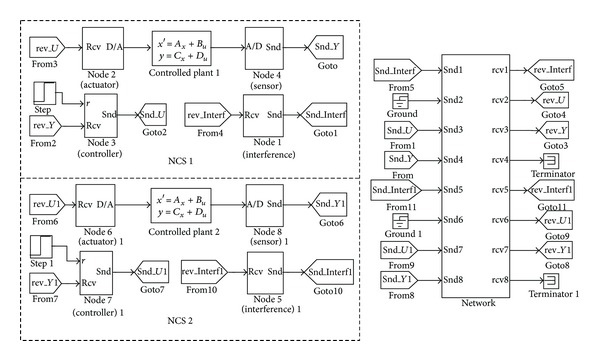
Two independent NCSs based on one 100 M switch.

Finally, in order to analyze whether the two NCSs whose control loops are closed through different ports on the same switch have influence on each other, we establish two independent NCSs (NCS 1 and NCS 2) by only one 100 M switched Ethernet, as shown in Figure 8. The controlled plants (Plant 1 and Plant 2), the structures, the controller parameters, and the network topologies of the two NCSs are all the same as those of the NCS in Figure 6.

After simulation, the results show that the step responses of the two NCSs are exactly the same as those of the NCS based on single-level 100 M switched Ethernet, which proves that the different NCSs established by one 100 M switch have no effect on each other if the source ports and destination ports of different control loops are completely different.

## 5. Conclusions

The delay induced by control networks is the most important factor to affect the performance of an NCS. In this paper, an NCS based on 100 M commercial switched Ethernet is proposed to solve the problems induced by the delay, provided that there is no packet dropout in the communication process. The delay induced by the single-level 100 M switched Ethernet is less than the time requirements of all classes of control systems, and the performance of an NCS based on a single-level 100 M switched Ethernet is the same as that of the nonnetwork control system, so it is feasible to apply the single-level 100 M switched Ethernet to all classes of control systems; however, the delay induced by the multiple-level 100 M switched Ethernet may meet the time requirements of control systems if the number of levels and the number of nodes connecting to the 100 M switches are set properly. Furthermore, we find that more than one NCS can be established by only one switch and they have no effect on each other if the source ports and the destination ports of different control loops are completely different. The feasibility and superiority of an NCS based on 100 M switched Ethernet are proved in this paper, and we hold that this class of the NCSs can be widely used in the fields of industrial automation.

## Figures and Tables

**Table 1:** The performance indexes of the control systems.

Network environments	Overshoot	Settling time/s
Nonnetwork	28.315%	0.304
100 M	42.111%	0.306
10 M	62.552%	0.382
CAN	94.111%	0.642
